# Anaerobic ammonium oxidation and its contribution to nitrogen removal in China’s coastal wetlands

**DOI:** 10.1038/srep15621

**Published:** 2015-10-23

**Authors:** Lijun Hou, Yanling Zheng, Min Liu, Xiaofei Li, Xianbiao Lin, Guoyu Yin, Juan Gao, Fengyu Deng, Fei Chen, Xiaofen Jiang

**Affiliations:** 1 State Key Laboratory of Estuarine and Coastal Research, East China Normal University, Shanghai 200062, China; 2 College of Geographical Sciences, East China Normal University, Shanghai 200241, China

## Abstract

Over the past several decades, human activities have caused substantial enrichment of reactive nitrogen in China’s coastal wetlands. Although anaerobic ammonium oxidation (anammox), the process of oxidizing ammonium into dinitrogen gas through the reduction of nitrite, is identified as an important process for removing reactive nitrogen, little is known about the dynamics of anammox and its contribution to nitrogen removal in nitrogen-enriched environments. Here, we examine potential rates of anammox and associate them with bacterial diversity and abundance across the coastal wetlands of China using molecular and isotope tracing techniques. High anammox bacterial diversity was detected in China’s coastal wetlands and included *Candidatus Scalindua, Kuenenia, Brocadia*, and *Jettenia*. Potential anammox rates were more closely associated with the abundance of anammox bacteria than to their diversity. Among all measured environmental variables, temperature was a key environmental factor, causing a latitudinal distribution of the anammox bacterial community composition, biodiversity and activity along the coastal wetlands of China. Based on nitrogen isotope tracing experiments, anammox was estimated to account for approximately 3.8–10.7% of the total reactive nitrogen removal in the study area. Combined with denitrification, anammox can remove 20.7% of the total external terrigenous inorganic nitrogen annually transported into China’s coastal wetland ecosystems.

Reactive nitrogen (Nr) production has increased over the past several decades^1^,^2^. Global Nr enrichment is one of the main emerging environmental problems of the twenty-first century^3^,^4^,^5^. Nr is now entering Earth’s land surface at more than twice its natural rate due to industrial production of nitrogen fertilizer and combustion of fossil fuels^2^,^4^. On a global basis, approximately 160 teragrams (Tg) of anthropogenic nitrogen are added to the Earth’s surface ecosystem each year^4^ in efforts to sustain food production for an increasing population and as parts of other social and economic human activities^6^. Approximately 20–30% of the anthropogenic nitrogen produced during fossil fuel combustion and food production and consumption is transported to estuarine and coastal marine systems via river flow^3^,^7^. This Nr pollution presents a serious threat to the environmental quality and health of these ecosystems in many areas of the world^8^,^9^, as increasing nitrogen input into estuarine and coastal marine systems is an important driver of water pollution (e.g., coastal eutrophication, hypoxia, harmful algae blooms, and seawater acidification)^9^,^10^.

China is seriously affected by Nr pollution^7^,^11^. Over the past century, Nr production in mainland China has increased from approximately 9.2 to 56 Tg per year^11^, with an annual growth rate of 1.8%. Since the 1950s, anthropogenic Nr creation from agriculture-associated biological nitrogen fixation, industrial nitrogen fixation, and fossil fuel combustion has exceeded natural Nr creation from lightning and biological nitrogen fixation in forests and grasslands^11^. Over the past three decades (1980–2010), anthropogenic nitrogen production was exponentially linked to gross national production, as well as to gross agricultural and industrial production (Supplementary Fig. S1). These strong associations imply that the input of anthropogenic nitrogen has greatly contributed to food and energy production in China. However, a large fraction of this human-produced nitrogen is delivered to atmospheric and aquatic environments^12^ due to low nitrogen-use efficiency by fertilized crops and other organisms. Nearly 20% of the total lost Nr, mostly in the form of nitrate, is transported into estuarine and coastal ecosystems by riverine discharge and atmospheric deposition^11^. As a result, the estuaries and coastal seas of China have become highly Nr-enriched ecosystems, characterized by extreme hyper-eutrophication and algal bloom formation (Supplementary Fig. S2). An improved understanding of nitrogen transformation and fate on a national scale is required to develop strategies to protect the water quality and health of these estuarine and coastal environments.

Anaerobic ammonium oxidation (anammox), the process of oxidizing ammonium through the reduction of nitrite, removes Nr permanently from aquatic ecosystems and returns it to the atmosphere as nitrogen gas^13^, as does denitrification. Anammox was first discovered in a wastewater treatment plant (WWTP) in the Netherlands in 1995^14^. Since that discovery, this process has been detected in various natural ecosystems, including freshwater sediments, marine sediments, and anaerobic water columns^13^,^15^,^16^,^17^,^18^,^19^,^20^,^21^. The anammox process has also been observed in the coastal wetlands of China^21^,^22^. However, these studies focused on local and small-scale regions. Gaps remain in our knowledge of the anammox process, including its association with anammox bacterial dynamics and its contribution to nitrogen loss across the coastal wetlands of China. To fill in these knowledge gaps, sediments (0–5 cm deep) were collected from the coastal wetlands of China in January and in August 2014 (Fig. 1), and molecular and isotope tracing experiments were conducted to obtain data on anammox bacteria and activity. This work examines for the first time the latitudinal distributions of anammox bacteria with regard to community composition, biodiversity, and activity, providing novel insights into the microbial nitrogen cycle in coastal wetlands.

## Results

### Anammox Rates

Anammox rates were measured at all sampling sites along the coastal wetlands of China using sediment slurry incubation experiments combined with a ^15^N isotope-labeling technique^23^,^24^. We conducted three independent groups of sediment slurry incubation experiments to quantify the rates of anammox and its contribution to N_2_ production, spiking each group with ^15^NH_4_^+^, ^15^NH_4_^+^ + ^14^NO_3_^−^, or ^15^NO_3_^−^. No significant production of isotope-labeled dinitrogen gas, including ^29^N_2_ and ^30^N_2_, was detected from the incubations with ^15^NH_4_^+^ alone in any of the collected sediment samples (Supplementary Fig. S3a), indicating complete consumption of background nitrate and nitrite in the sediment slurries during pre-incubation. With the addition of ^15^NH_4_^+^ + ^14^NO_3_^−^, significant production of ^29^N_2_ was detected in all sediment samples, but no significant production of ^30^N_2_ was observed (Supplementary Fig. S3b). The results from these two groups of experiments corroborated the occurrence of anammox at all sampling sites. By contrast, significant production of both ^29^N_2_ and ^30^N_2_ was detected from incubations containing added ^15^NO_3_^−^ (Supplementary Fig. S3c). Therefore, rates of anammox and its contributions to N_2_ production were quantified from the experiments in which ^15^NO_3_^−^ was added^15^,^25^.

Potential rates of anammox obtained in the sediment slurry incubation experiments ranged from 1.8 to 10.4 μmol N kg^−1^ d^−1^ (Fig. 2a). Anammox rates were significantly greater in summer than in winter (one-way ANOVA, *p* < 0.05), with respective average values of 7.0 ± 1.4 μmol N kg^−1^ d^−1^ and 3.2 ± 0.8 μmol N kg^−1^ d^−1^ (Supplementary Fig. S4). Significantly lower rates of anammox were recorded at coastal wetland sites S1 to S6 than at sites S7 to S11 (one-way ANOVA, *p* < 0.05), with average values of 5.9 ± 2.3 μmol N kg^−1^ d^−1^ and 4.3 ± 1.9 μmol N kg^−1^ d^−1^, respectively (Supplementary Fig. S5). Among all measured environmental variables (Supplementary Table S1), winter anammox rates were significantly related (Pearson’s correlation analyses) to temperature (*R* = 0.752, *p* = 0.008), nitrite (*R* = 0.667, *p* = 0.024), and ammonium (*R* = 0.833, *p* = 0.002), whereas summer anammox rates were significantly correlated with temperature (*R* = 0.607, *p* = 0.048), nitrate (*R* = 0.649, *p* = 0.030), nitrite (*R* = 0.692, *p* = 0.018), and organic carbon (*R* = 0.717, *p* = 0.012) (Supplementary Table S2). The estimated contributions of anammox to total N_2_ production varied from 3.8 to 10.7% in winter and from 4.5 to 5.8% in summer (Fig. 2b); the remainder would be attributed to denitrification.

### Composition, Diversity, and Abundance of Anammox Bacteria

To further decipher the microbial mechanisms regulating anammox, the community composition, diversity, and abundance were examined based on the 16S rRNA gene of anammox bacteria. Phylogenetic analysis of the 16S rRNA genes indicated the presence of four known anammox bacterial genera, *Candidatus Scalindua, Kuenenia, Brocadia*, and *Jettenia*, as well as a novel cluster (Supplementary Fig. S6). Weighted UniFrac PCoA analysis with the 16S rRNA gene sequences showed no significant seasonal shift in the community composition or diversity of the anammox bacteria at most of the sampling sites (except for sites S2 and S4; Supplementary Fig. S7). However, a distinctive latitudinal differentiation was observed in the community composition and diversity of the anammox bacteria along the coastal wetlands of China (Fig. 3). The community of anammox bacteria at sites S1 to S6 was mainly dominated by *Scalindua*, which accounted for >90% of the sequences (Supplementary Fig. S8). By contrast, significant shifts were recorded in the compositions of the anammox bacteria communities at sites S7 to S11, which consisted mainly of *Scalindua, Kuenenia*, and (or) *Brocadia*. Potential relationships between these anammox bacterial distributions and environmental factors were examined by weighted canonical correspondence analysis (CCA). Of all the environmental factors investigated, only temperature had a significant effect on the anammox bacterial community distribution (*p* = 0.002 for winter; *p* = 0.038 for summer; Supplementary Fig. S9).

The abundance of anammox bacteria in the coastal wetland sediments of China was estimated with qPCR assays based on the 16S rRNA gene. The quantity of anammox bacterial 16S rRNA genes observed ranged between 1.17 × 10^7^ and 4.25 × 10^7^ copies g^−1^ dry sediment (Fig. 4). Anammox bacterial abundance was characterized by significant spatiotemporal heterogeneity at the study area (one-way ANOVA, *p* < 0.05). The abundance of anammox bacteria was generally higher in summer than in winter, with average abundances of 2.87 ± 0.8 × 10^7^ copies g^−1^ dry sediment in summer and 2.05 ± 0.6 × 10^7^ copies g^−1^ dry sediment in winter (Supplementary Fig. S10). Additionally, the spatial distribution of anammox bacterial abundance was characterized by an increasing trend from high to low latitudes (Supplementary Fig. S11). Pearson’s correlation analyses revealed that the anammox bacterial abundance was related significantly to temperature (*R* = 0.862, *p* < 0.0001) and the amounts of sedimentary ammonium (*R* = 0.814, *p* = 0.002) and nitrite (*R* = 0.795, *p* = 0.006) in winter, whereas it was closely related to temperature (*R* = 0.790, *p* = 0.004) and the amounts of sedimentary nitrate (*R* = 0.802, *p* = 0.004) and nitrite (*R* = 0.856, *p* < 0.0001) in summer (Supplementary Table S3).

### Other Nitrogen Transformation Processes

Potential rates of related nitrogen cycling processes, including denitrification, nitrification and dissimilatory nitrate reduction to ammonium (DNRA), were determined to examine their potential links to anammox (Fig. 5). Denitrification rates in sediments in the study area ranged from 20.8 to 78.9 μmol N kg^−1^ d^−1^ in winter. Significantly higher denitrification rates were observed in summer (one-way ANOVA, *p* < 0.05), with values of 87.8–182.1 μmol N kg^−1^ d^−1^. In winter, potential nitrification rates in sediments varied between 10.9 and 19.6 μmol N kg^−1^ d^−1^. However, the relatively higher rates of nitrification in summer ranged from 12.9 to 29.3 μmol N kg^−1^ d^−1^ (one-way ANOVA, *p* < 0.05). Compared to denitrification and nitrification, DNRA showed relatively low rates, with values of 3.5–10.1 μmol N kg^−1^ d^−1^ in winter and 11.4–19.5 μmol N kg^−1^ d^−1^ in summer. Pearson’s correlation analyses showed that summer anammox rates were significantly correlated with denitrification rates (*R* = 0.870, *p* = 0.0004), whereas in winter, anammox rates correlated with nitrification rates (*R* = 0.745, *p* = 0.008) (Fig. 6). Anammox and DNRA rates did not correlate significantly in the study area (*p* > 0.05).

## Discussion

Both molecular and isotope-tracing experiments corroborated the occurrence of anammox in the coastal wetland sediments of China. The anammox process is mediated by chemolithoautotrophic bacteria affiliated with the order *Brocadiales*, part of the phylum Planctomycetes^26^. At present, five genera of anammox bacteria (*Candidatus Brocadia, Kuenenia, Scalindua, Anammoxoglobus* and *Jettenia*) have been identified from different wastewater treatment systems^26^. Using molecular techniques, the biodiversity of anammox bacteria was recently studied in various natural ecosystems and determined to be relatively low for marine, riverine, and lacustrine ecosystems^27^; community compositions in these environments were restricted mainly to *Scalindua* or *Brocadia*^27^,^28^,^29^,^30^. By contrast, a relatively high biodiversity of anammox bacteria, including *Candidatus Scalindua, Kuenenia, Brocadia*, and *Jettenia* (based on the 16S rRNA gene), was detected in our study area. These results are similar to the high biodiversity of anammox bacteria observed in other estuarine and coastal wetlands^19^,^31^,^32^. Therefore, this comparison implies that high anammox bacterial biodiversity may occur in the transitional zones between terrestrial and marines ecosystems, where diverse biotopes and eco-niches exist in complex ecotones. Although the biodiversity of anammox bacteria did not show a statistically significant seasonal shift, it was characterized by a distinctive latitudinal differentiation along the coastal wetlands of China. Numerous studies have reported that salinity is a key environmental factor shaping the diversity and biogeographical distribution of anammox bacteria^21^,^27^,^28^,^33^. However, in our study area, the distribution of the anammox bacterial community was not significantly related to salinity, but it did correlate with temperature (Supplementary Fig. S9). Compared with the high latitude sites, low latitude sites contained more diverse communities of anammox bacteria. These results imply that temperature is a key environmental factor shaping the biogeographical distribution and diversity of the anammox bacterial community in the coastal wetlands of China. Therefore, differences in the eco-physiology of these anammox bacteria might be a main mechanism defining the geographical distribution pattern of the anammox bacterial community. The latitudinal distribution pattern observed in the present study emphasizes the importance of temperature in controlling the biogeographical distribution of anammox bacterial community structure and diversity at a larger spatial scale.

Anammox bacterial abundance demonstrated significant spatial and seasonal fluctuations in the study area. Similarly, temperature was a key environmental variable controlling the spatio-temporal heterogeneity of anammox bacteria abundance in sediments (Pearson’s correlation analyses; Supplementary Table S3). The significant correlation between nitrite and anammox bacteria abundance implies that substrate availability is also an important factor affecting the number of anammox bacteria in coastal wetland sediments^15^,^34^,^35^. Nitrite may help limit anammox bacterial population development in the coastal wetland sediments of China. However, a seasonal shift was observed in the underlying mechanism controlling substrate availability. In winter, nitrite may derive from nitrification. Significant associations between sedimentary nitrite content and nitrification rate (*R* = 0.609, *p* = 0.047) as well as sedimentary ammonium content (*R* = 0.710, *p* = 0.014) support this conclusion. By contrast, nitrite is more likely produced from denitrification in summer, as supported by the significant relationships of sedimentary nitrite content with denitrification rate (*R* = 0.700, *p* = 0.016) and sedimentary nitrate content (*R* = 0.829, *p* = 0.002).

Anammox rates and contribution to nitrogen removal were examined in the coastal wetland sediments of China. Our isotopic tracing experiments revealed rates of anammox that are comparable to the values reported in other estuarine and coastal wetland sediments (Table 1). Anammox rates in the coastal wetland sediments of China were characterized by substantial spatial and seasonal heterogeneity, which might relate to sampling site biotic and abiotic factors. In the present study, anammox rates were not significantly related to the anammox bacterial community composition and structure (*p* > 0.05; Supplementary Fig. S12) but did correlate with their abundance (*R* = 0.852, *p* = 0.0008 for winter; *R* = 0.633, *p* = 0.036 for summer; Supplementary Fig. S13). This result demonstrates that anammox bacterial abundance, more than composition and diversity, predicts anammox activity^31^. In addition, anammox rates were significantly and positively correlated with temperature (*R* = 0.752, *p* = 0.008 for winter; *R* = 0.607, *p* = 0.048 for summer). High temperature may favor the metabolism of anammox bacteria and thus promote rates of anammox^22^,^36^. The relationships between anammox rates and sedimentary nitrite concentrations (*R* = 0.667, *p* = 0.024 for winter; *R* = 0.692, *p* = 0.018 for summer) imply that substrate availability is a limiting factor affecting the activity of anammox bacteria in the coastal wetland sediments of China.

Anammox rates were compared to nitrification, denitrification and DNRA rates to elucidate the potential links among these nitrogen transformation processes. Anammox rates were not significantly related to DNRA rates but did relate to nitrification and denitrification rates (Fig. 6). These correlation analyses reflected that anammox might be closely associated with nitrification and denitrification in the study area. Interestingly, the links between anammox and nitrification and denitrification shifted seasonally. In winter, anammox correlated predominantly with nitrification, suggesting partial nitrification as a primary source of nitrite for winter anammox reactions. This conclusion is also supported by the significant relationships of anammox rates with sedimentary ammonium (*R* = 0.833, *p* = 0.002) and nitrite (*R* = 0.667, *p* = 0.024) contents. By contrast, summer anammox shifted to coupling with denitrification. During the interaction between anammox bacteria and denitrifiers, denitrification was likely a primary source of nitrite for anammox in the coastal wetland sediments of China^34^. The strong linkage of both processes is supported by the significant correlations of anammox rates with sedimentary nitrate (*R* = 0.649, *p* = 0.030) and organic carbon (*R* = 0.717, *p* = 0.012) contents. In summer, more nitrite was generated as an intermediate product by enhanced denitrification (Supplementary Fig. S14). As a result, the development of anammox bacteria was stimulated (Supplementary Table S3) and enhanced anammox rates in the warm season (Supplementary Fig. S13b). Although the DNRA process can also produce nitrite as an intermediate product^36^ and thereby act as an alternative source of substrate for anammox bacteria^16^,^18^, no significant relationship was detected between anammox and DNRA rates (*p* > 0.05), implying that DNRA-produced nitrite contributes minimally to the metabolic activity of anammox bacteria in nitrogen-enriched coastal wetlands where denitrification and nitrification are active^37^.

We estimated that the anammox process, on average, contributed approximately 5.7 ± 1.2% to total nitrogen loss from the coastal wetland sediments of China; this approximation is comparable to that reported in rivers^38^, lakes^39^,^40^, constructed wetlands^41^, and other estuarine and coastal environments^23^,^24^,^42^,^43^,^44^,^45^,^46^,^47^,^48^,^49^ but lower than that generally observed in marine ecosystems^15^,^16^,^17^,^18^. If the average rate of anammox is extrapolated to the entire study area (5.8 × 10^6^ ha), approximately 2.81 ± 1.1 × 10^4^ t N can be removed annually from the coastal wetland ecosystem by this process, based on dry sediment bulk density (1.88 g cm^–^^3^) in the study area (Supplementary Fig. S15). This removal accounts for approximately 1.2 ± 0.5% of the total multi-annual average terrigenous inorganic nitrogen (2.4 × 10^6^ t N) transported into the coastal wetlands of China. If denitrification is also included, both processes can remove approximately 4.98 ± 1.87 × 10^5^ t N from the study area on an annual basis, which accounts for approximately 20.7 ± 7.8% of the total external terrigenous inorganic nitrogen. Furthermore, assuming that the coastal wetlands of China have not been destroyed by coastal development in recent decades, up to 49.3 ± 18.6% of terrigenous inorganic nitrogen could be removed by both denitrification and anammox, which would greatly benefit the ecological health of China’s coastal seas.

Taken as a whole, this study is the first to investigate anammox rates, the contribution of anammox rates to nitrogen removal, and the dynamics of anammox bacteria in the coastal wetlands of China on a national scale. High biodiversity of anammox bacteria was observed in the coastal wetlands and included *Candidatus Scalindua, Kuenenia, Brocadia*, and *Jettenia*. Compared to denitrification, anammox contributed approximately 5.7 ± 1.2% of the total loss of reactive nitrogen at the study area. The present study highlights the importance of temperature in regulating the latitudinal distribution of the anammox bacterial community composition, diversity, abundance, and activity in natural environments on a large spatial scale. This study also suggests the significance of protecting coastal wetlands for the sustainable development of society and the economy in China’s coastal zone.

## Methods

### Study Area

The Chinese coastal zone covers an area of more than three million square kilometers with an 1800-km coastline stretching across tropical, subtropical and temperate zones^50^. Coastal development in China has contributed greatly to the national economy. The gross domestic productivity (GDP) in the coastal regions accounted for 50% of China’s total GDP before 1978 and increased to 60% between 1978 and 2010^51^. Meanwhile, China’s coastal population increased from 260 million in 1954, to 400 million in 1978, and 590 million in 2010^51^. The continuing increase in population coupled with economic growth, rapid urbanization, and infrastructure development in the coastal zone of China have caused significant changes in coastal wetland ecosystems^52^. Over the past several decades, China’s coastal wetlands have suffered tremendous loss due to increased threats and pressures on wetlands by the increasing population and rapid economy growth. From 1950 to 2014, the area of coastal wetlands decreased from 13.81 × 10^6^ ha to 5.8 × 10^6^ ha, with a total loss rate of 58%^52^. Numerous factors endanger the existence of coastal wetlands, such as land demands by a large human population, a lack of understanding of coastal wetland values, a misguided reclamation policy, and a lack of environmental laws and regulations. Among them, reclamation and infrastructure construction were the primary causes of wetland loss, accounting for 70–82% of the total loss^53^.

### Sample Collection

**Figure 1:**
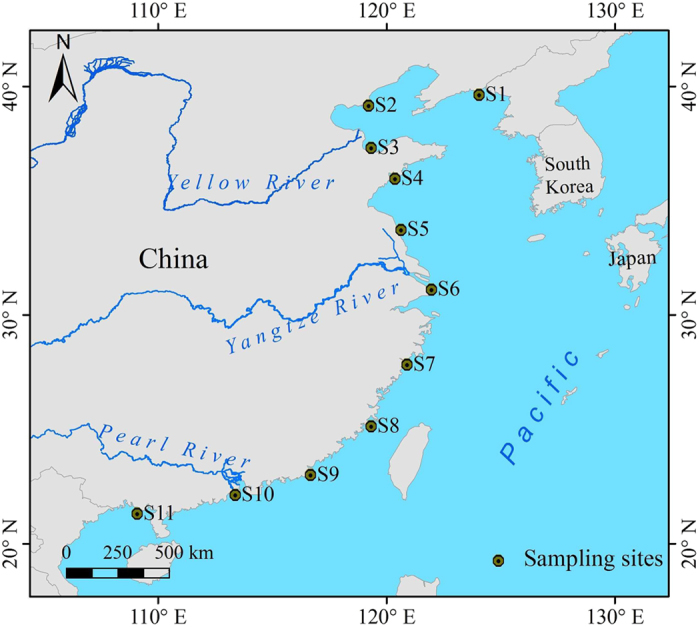
Study area and sampling sites. In this study, eleven sampling sites S1-S11 were selected along the coastal wetlands of China. The map was created with ArcGIS 10.1.

**Figure 2:**
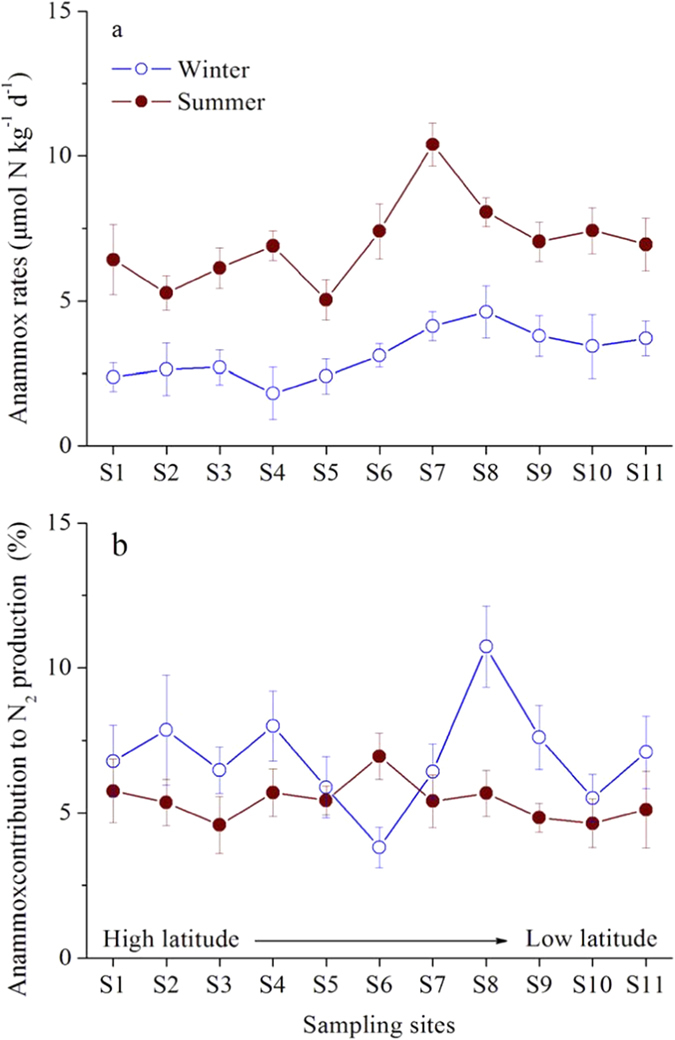
Spatiotemporal variations of anaerobic ammonium oxidation (anammox) rates (a) and associated contribution (b) to nitrogen loss at each sampling site. Vertical bars indicate standard error (n = 3).

**Figure 3:**
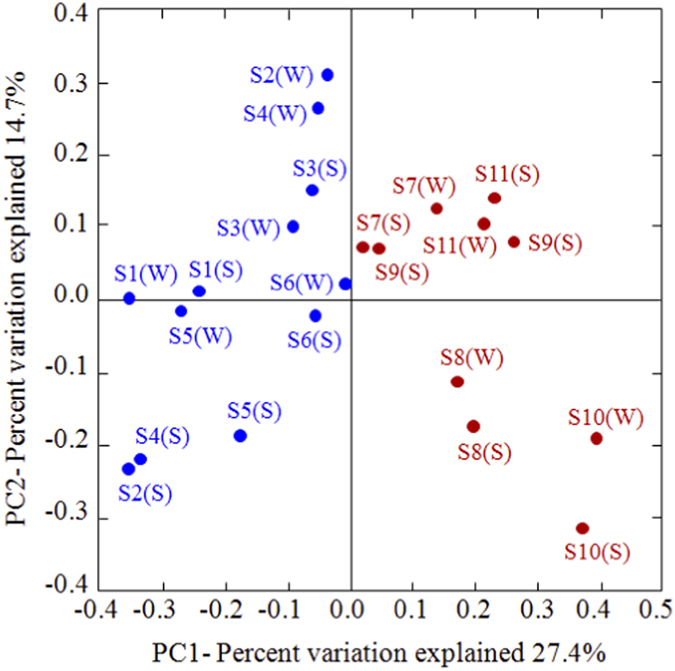
UniFrac weighted PCoA analysis of anaerobic ammonium oxidation (anammox) bacterial assemblages as revealed by the 16S rRNA gene sequences. S and W in parentheses represent summer and winter samples, respectively.

**Figure 4:**
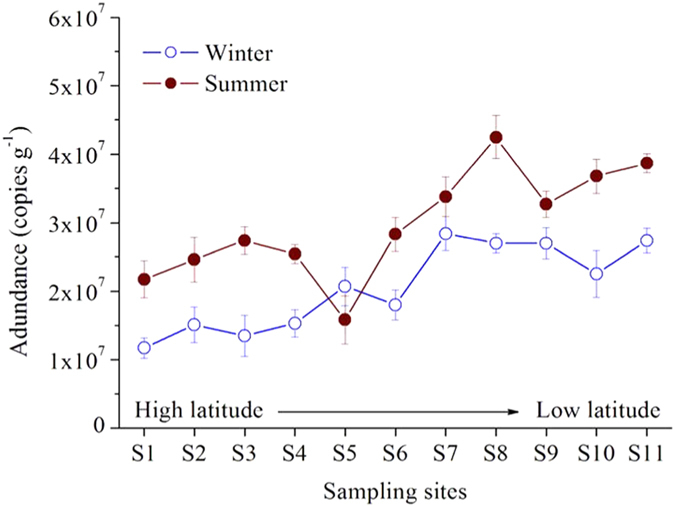
Spatiotemporal variations of anaerobic ammonium oxidation (anammox) bacterial abundance targeting on the 16S rRNA gene in China’s coastal wetland sediments. Vertical bars indicate standard error (n = 3).

**Figure 5:**
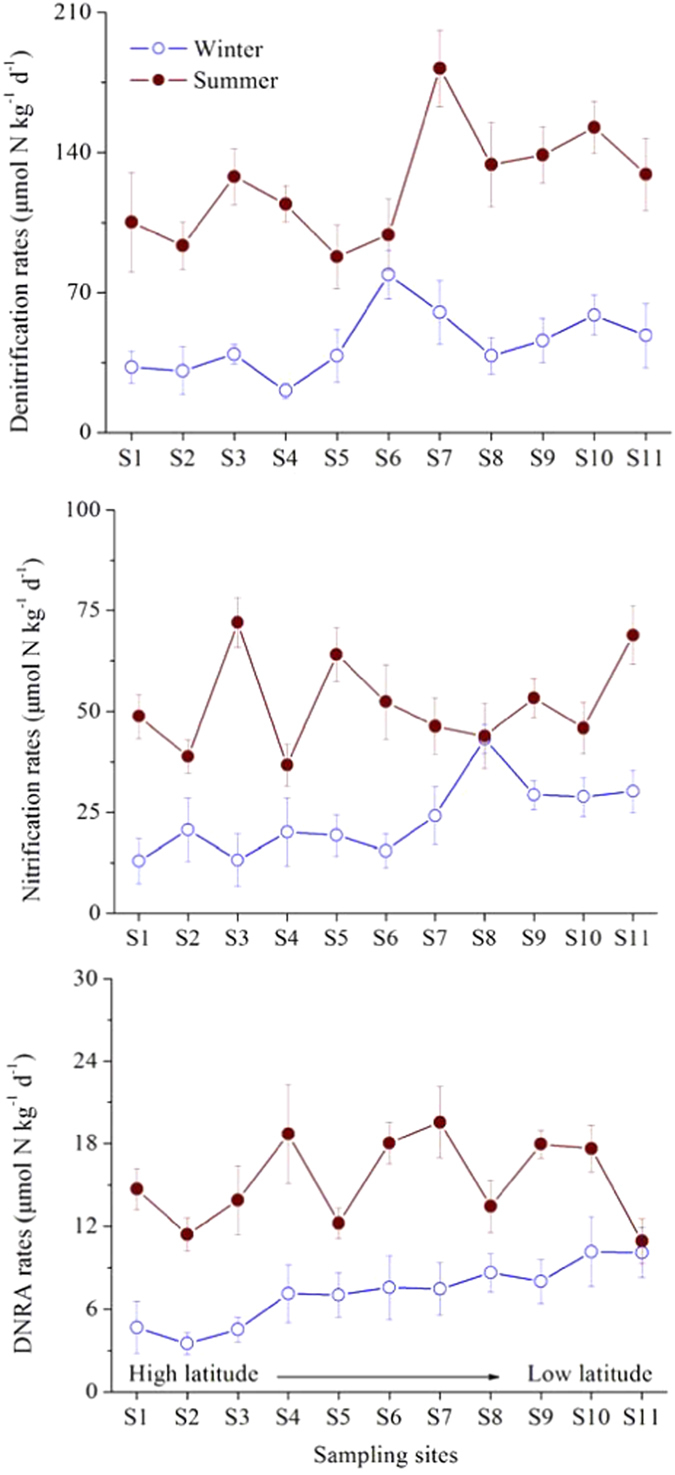
Spatiotemporal variations of dentirification, nitrification, and dissimilatory nitrate reduction to ammonium (DNRA) rates at each sampling site. Vertical bars indicate standard error (n = 3).

**Figure 6:**
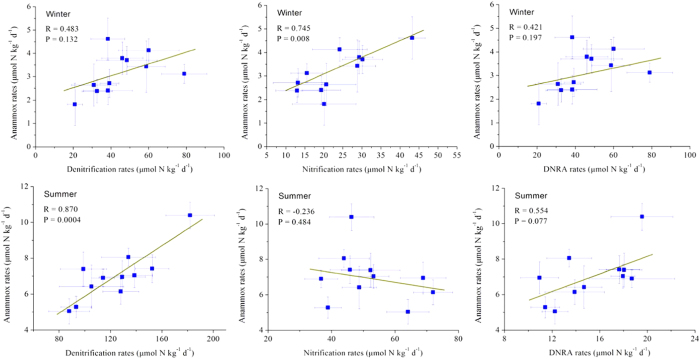
Pearson’s correlations of anaerobic ammonium oxidation (anammox) rates with denitrification, nitrification and dissimilatory nitrate reduction to ammonium (DNRA) rates in China’s coastal wetlands. Vertical and horizontal bars indicate standard error (n = 3).

In this study, eleven sampling sites were selected along the coastal wetlands of China (Fig. 1). Field surveys were carried out in January and in August 2014. Triplicate sediment samples (0–5 cm deep) were collected from each site with PVC tubes. Sediment cores were stored in sterile plastic bags, sealed and transported to the laboratory on ice within 4–8 h. The sediment of each core was mixed thoroughly under helium to form one composite sample. One portion of the homogenized sediment was incubated immediately to measure nitrogen transformation rates, and another portion was examined for sediment physiochemical characteristics. Subsamples were also preserved at −80 °C for DNA extraction and molecular analysis.

### Analysis of Sediment Characteristics

Sediment temperature was measured *in situ* with a portable electronic thermometer at each site. Sediment salinity and pH were measured using a YSI Model 30 salinity meter and a Mettler-Toledo pH meter, respectively, after the sediments were mixed with deionized water free of CO_2_ at a sediment/water volume ratio of 1:2.5^22^. Sediment grain size was measured using a LS 13 320 Laser grain sizer. Organic carbon (OC) was determined by a CHN elementary analyzer (VVarioELIII, Elementary, Germany) after removing carbonate by leaching with 0.1 mol L^−1^ HCl^22^. Exchangeable ammonium (NH_4_^+^-N), nitrite (NO_2_^−^-N), and nitrate (NO_3_^−^-N) were extracted from fresh sediments with 2 mol L^−1^ KCl and measured spectrophotometrically on a continuous-flow nutrient analyzer (SAN plus, *Skalar Analytical* B.V., the Netherlands), with detection limits of 0.5 μmol L^−1^ for NH_4_^+^-N and 0.1 μmol L^−1^ for NO_2_^−^-N and NO_3_^−^-N^22^. Sulfide was measured using an Orion Sure-flow^®^ combination silver-sulfide electrode (Thermo Scientific Orion)^54^, with a detection limit of 0.09 μmol L^−1^. All sediment physiochemical parameters were analyzed in triplicate. Detailed information on sediment characteristics is given in Supplementary Table S1.

### DNA Isolation, PCR, Cloning, Sequencing, and Phylogenetic Analysis

Total genomic DNA was extracted from 0.25 g of each sediment sample using Powersoil^TM^ DNA Isolation Kits (MOBIO, USA) according to the manufacturer’s protocol. Nested PCR assays were conducted to detect the anammox bacterial 16S rRNA gene. The initial PCR reaction was conducted using PLA46f-1390r primers with a thermocycling program of 94 °C for 4 min followed by 30 cycles of 45 s at 95 °C, 50 s at 59 °C, 1 min 40 s at 72 °C, and a final 5 min extension cycle at 72 °C^22^. One μL of the 10-fold diluted PCR product was then used as a template for the second amplification with Amx368f-Amx820r primers using a thermocycling program of 94 °C for 4 min followed by 30 cycles of 45 s at 95 °C, 50 s at 59 °C, 1 min at 72 °C, and a final 5 min extension cycle at 72 °C^22^.

Appropriately sized fragments of the nested PCR products were separated by electrophoresis in 1% agarose gels and purified using the Gel Advance-Gel Extraction system (Viogene, China). The purified fragments were cloned by TOPO-TA cloning (Invitrogen, USA) in accordance with the manufacturer’s instructions. Clones were selected randomly for further analysis. The unique sequences obtained in this study for anammox bacteria are available in NCBI under accession numbers KT229750-KT230483. All of the sequences and their relatives obtained from NCBI were aligned using the ClustalX program (version 2.1)^55^. Sequences with 97% identity were grouped into one operational taxonomic unit (OTU) using the Mothur program (version 1.33.0, USA) (http://www.mothur.org/wiki/Main_Page) by the furthest neighbor approach. Phylogenetic trees were constructed by the neighbor-joining (NJ) method using MEGA software (version 5.03)^56^. The relative confidence of the tree topologies was evaluated by performing 1000 bootstrap replicates^57^.

### Real-Time Quantitative PCR Assay

Plasmids carrying an anammox bacterial 16S rRNA gene fragment were extracted from *Escherichia coli* hosts using a Plasmid Mini Preparation Kit (Tiangen, China) for standard curve construction. Plasmid DNA concentrations were measured with a Nanodrop-2000 Spectrophotometer (Thermo, USA). Triplicate samples and standard reactions were performed with primers AMX-808-F and AMX-1040-R on an ABI 7500 Sequence Detection System (Applied Biosystems, Canada)^22^. The 25 μL qPCR mixture contained 12.5 μL of Maxima SYBR Green/RoxqPCR Master Mix (Fermentas, Lithuania), 1 μL of each primer (10 μmol L^−1^) and 1 μL template DNA. All reactions were performed in 8-strip thin-well PCR tubes with ultraclear cap strips (ABgene, United Kingdom). PCR amplification was performed as follows: 50 °C for 2 min, 95 °C for 10 min, followed by 45 cycles of 30 s at 95 °C, 30 s at 55 °C, 30 s at 72 °C. The specificity of the Q-PCR amplification was determined by melting curve and gel electrophoresis. In all experiments, negative controls containing no template DNA were subjected to the same qPCR procedure to detect and exclude any possible contamination or carryover.

Melting-curve analyses of the anammox bacterial 16S rRNA revealed a single peak at 84.3 °C, corroborating that fluorescent signals were derived from specific PCR products in the process of qPCR quantifications. Standard curves were generated from plasmids containing cloned 16S rRNA gene fragments by plotting the threshold cycle (Ct) versus the log_10_ value of the gene copy number. A significant linear relationship (*R*^2^ = 0.99) was obtained over 6 orders of magnitude of the standard plasmid DNA concentration (2 × 10^1^ to 2 × 10^7^ copies μL^−1^), indicating high primer hybridization and extension efficiency.

### Anammox and Denitrification Rates

Sediment slurry experiments were conducted to measure rates of anammox and denitrification with a nitrogen isotope tracing method. Detailed information on slurry experiments is also described in refs 23,24. In brief, slurries were prepared with fresh sediment and helium-purged overlying water from each site at a sediment/water volume ratio of 1:5. The resulting slurries were transferred into a series of 12-mL glass vials (Exetainer, Labco, High Wycombe, Buckinghamshire, UK) under a helium atmosphere and pre-incubated for 24 h at near *in situ* temperature to eliminate residual nitrate, nitrite and oxygen. The vials were then divided into three groups, which were spiked (through the septa of each vial) with helium-purged stock solutions of (1) ^15^NH_4_^+^, (2) ^15^NH_4_^+^ + ^14^NO_3_^−^, or (3) ^15^NO_3_^−^. The final concentration of ^15^N in each vial was approximately 100 μmol L^−1^. Slurry incubation was blocked by injecting 300 μl of 50% ZnCl_2_ solution into each vial after 8 h. The incubation time was determined based on a test experiment that showed a linear increase in N_2_ production during an 8-h incubation (Supplementary Fig. 3S). Concentrations of ^29^N_2_ and ^30^N_2_ produced during the incubation were measured by membrane inlet mass spectrometry (MIMS)^22^. Rates of both anammox and denitrification and their respective contribution to total N_2_ production were calculated using the methods given in refs 15, 25.

### Dissimilatory Nitrate Reduction to Ammonium (DNRA) Rates

DNRA rates in slurry experiments were measured with a nitrogen isotope tracing method as described in ref. 54. Slurries were made with fresh sediment and helium-purged site water at a sediment-water volume ratio of 1:5 and transferred into 12-mL helium-flushed glass vials (Exetainer, Labco, High Wycombe, Buckinghamshire, UK). Subsequently, they were pre-incubated to eliminate residual nitrate, nitrite, and oxygen at near *in situ* temperature. The slurry vials were then spiked with ^15^NO_3_^−^ (final concentration ca. 100 μmol L^−1^), and one half of the replicates were designated as initial samples and preserved with 300 μL of 50% ZnCl_2_. The remaining slurries were shaken (200 rpm), incubated for approximately 8 h at near *in situ* temperature, and preserved as final samples with ZnCl_2_ solution, as described for initial samples. The concentrations of ^15^NH_4_^+^ produced via DNRA in the slurry incubations were analyzed by the Oxidation/MIMS (OX/MIMS) method^54^. Potential DNRA rates were estimated according to the changes in ^15^NH_4_^+^ concentration during the incubations^54^.

### Potential Ammonium Oxidation Rates

Potential nitrification rates were quantified in triplicate according to ref. 58. Briefly, 10 g of fresh sediment was added to 250-mL Erlenmeyer flasks containing 100 mL of overlying water from the site. NH_4_Cl (for ammonium) and KH_2_PO_4_ (for phosphate) were amended to the flasks at final concentrations of 300 μmol L^−1^ and 60 μmol L^−1^, respectively. The suspension was incubated in the dark at near *in situ* temperature with continuous shaking at 120 rpm. During the incubations, subsamples (2 mL) were harvested at 0, 4, 8, and 12 h and were centrifuged, filtered and frozen immediately for nitrate (plus nitrite) analysis. Potential nitrification rates were estimated based on the linear increase of nitrate concentrations in suspension during incubation^58^.

### Statistical Analyses

The Mothur program (version 1.33.0) was used to generate rarefaction curves for the observed unique OTUs and to determine the biodiversity indicators (Shannon and Chao 1) for each constructed gene library. The coverage of each clone library (>90% in this study) was estimated by the percentage of the observed number of OTUs divided by the Chao1 estimate (Supplementary Table S4). The ecological distribution of anammox bacterial communities and their correlations with environmental factors were determined by Weighted UniFrac PCoA analysis and canonical correspondence analysis (CCA), respectively, using CANOCO software^59^. Pearson correlation analyses were conducted with SPSS (version 11.5) to examine correlations among anammox bacterial diversity, abundance and environmental factors. In addition, a one-way ANOVA was performed to compare spatial and seasonal differences in nitrogen transformation rates.

## Additional Information

**How to cite this article**: Hou, L. *et al.* Anaerobic ammonium oxidation and its contribution to nitrogen removal in China's coastal wetlands. *Sci. Rep.* **5**, 15621; doi: 10.1038/srep15621 (2015).

## Supplementary Material

Supplementary Information

## Figures and Tables

**Table 1:** Rates of anaerobic ammonium oxidation (anammox) and associated contribution to nitrogen removal in China’s coastal wetlands and other estuarine and coastal ecosystems.

**Locations**	**Rates (μmol N m**^−**2**^ **h**^−**1**^)	**Contributions (%)**	**Refs**
New River Estuary	0.3–50.8^a^	<14	19
Seine Estuary	11.8–24.9^a^	3–8	20
Rander Fjord	19–55	5–24	23
Norsminder Fjord			
Thames Estuary	2.2–200	0.5–8	25
Cape Fear River Estuary	1.5–14.8^a^	3.8–14.6	31
Southeast coast of Queensland	0–84	0–9	34
West and south coast of Greenland	1–92	1–35	42
Gulf of Finland	10–30	10–15	43
Chesapeake Bay	0–1.6	0–22	44
Colne Estuary	0–157	<30	45
Plum Island Sound Estuary	2.9	<3	46
St. Lawrence Estuary	5.5	3	47
Providence River Estuary	0–16	0–4	48
Little Lagoon	0.2–0.5	2–4	49
China’s Coastal wetlands	2.0–11.4^a^	3.8–10.7	This study

^a^The unit is changed from μmol N g^−1^ h^−1^ to μmol N m^−2^ h^−1^, assuming dry sediment bulk density of 1.88 g cm^−3^.
