# Supplementary material for: Anaerobic ammonium oxidation and its contribution to nitrogen removal in China’s coastal wetlands
Source: Sci Rep. 2015 Oct 23;5:15621. doi: 10.1038/srep15621 (PMC4616045; doi:10.1038/srep15621)
Supplement: Supplementary Information [file srep15621-s1.pdf]

Supplementary Information for

Anaerobic ammonium oxidation and associated contribution to nitrogen removal in China's coastal wetlands

Lijun Hou*, Yanling Zheng, Min Liu*, Xiaofei Li, Xianbiao Lin, Guoyu Yin, Juan Gao, Fengyu Deng, Fei Chen & Xiaofen Jiang

*Corresponding authors: Lijun Hou and Min Liu

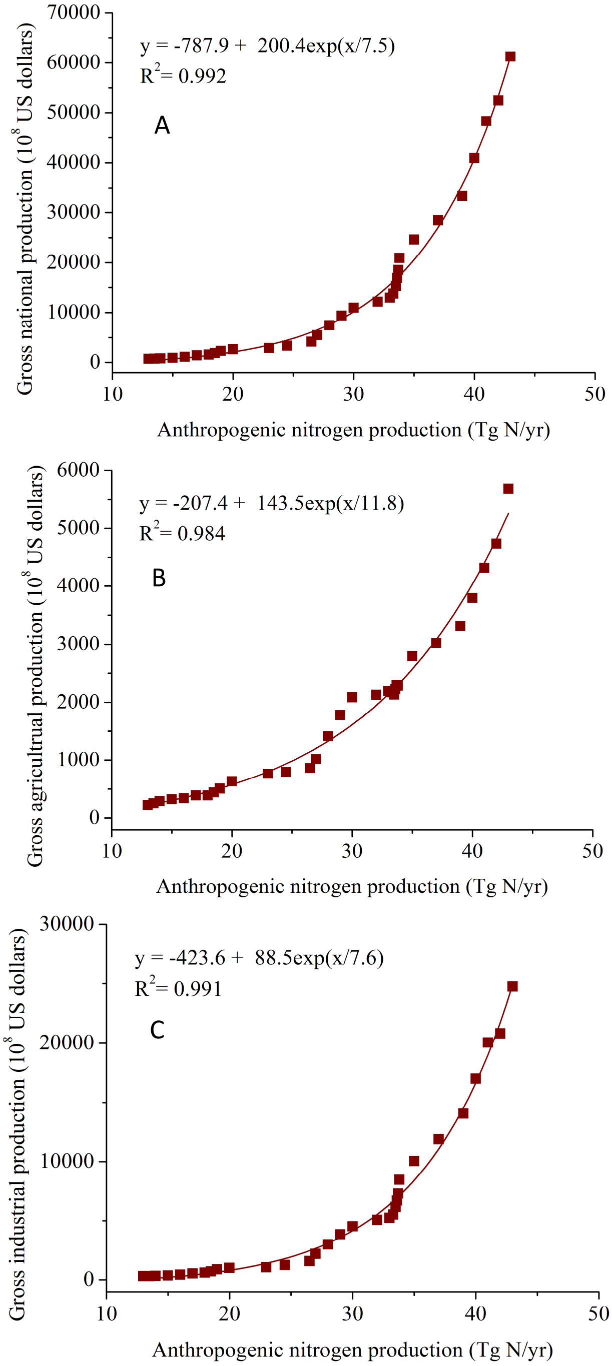

Supplementary Figure S1 Exponential relationships of anthropogenic nitrogen production and gross national production (a), gross agricultural production (b) and gross industrial production (c) in mainland China during the period of 1980 to 2010. The data on the anthropogenic nitrogen production are obtained from Ref 1, while the data on the gross national production, gross agricultural production and gross industrial production are obtained from China Statistical Yearbook (1980-2010).

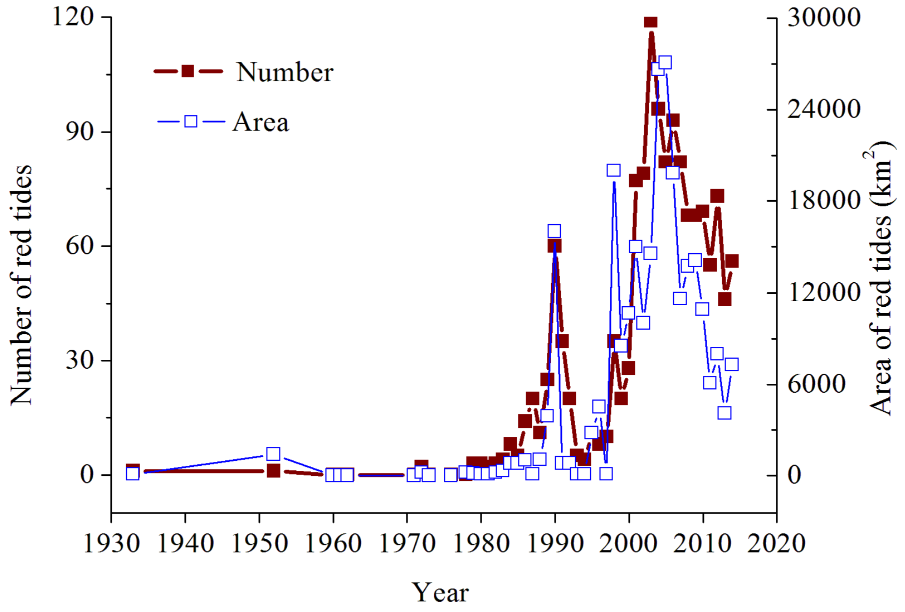

Supplementary Figure S2 Number and area of red tides recorded in China's coastal seas over the past several decades. The data on the number and area of red tides before 2000 are obtained from Ref 2, while the data after 2000 are obtained from China's Marine Environment Bulletin.

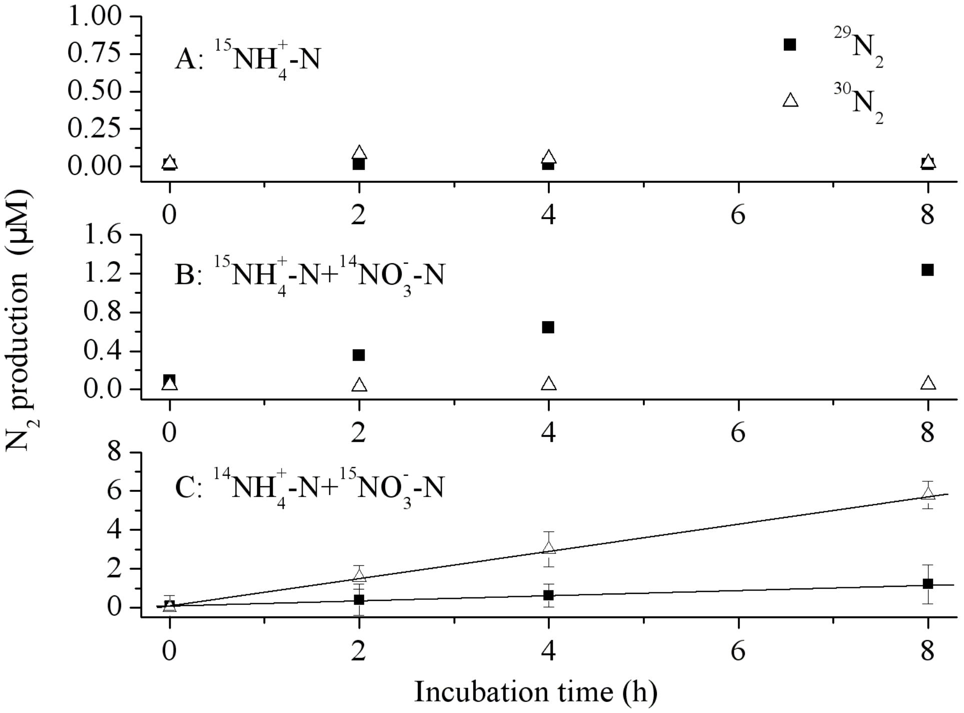

Supplementary Figure S3 Examples showing concentrations of $^{29}N_2$ and $^{30}N_2$ in sediment samples spiked with $^{15}NH_4^+$ (A), $^{15}NH_4^+$ + $^{14}NO_3^-$ (B) and $^{15}NO_3^-$ (C), respectively.

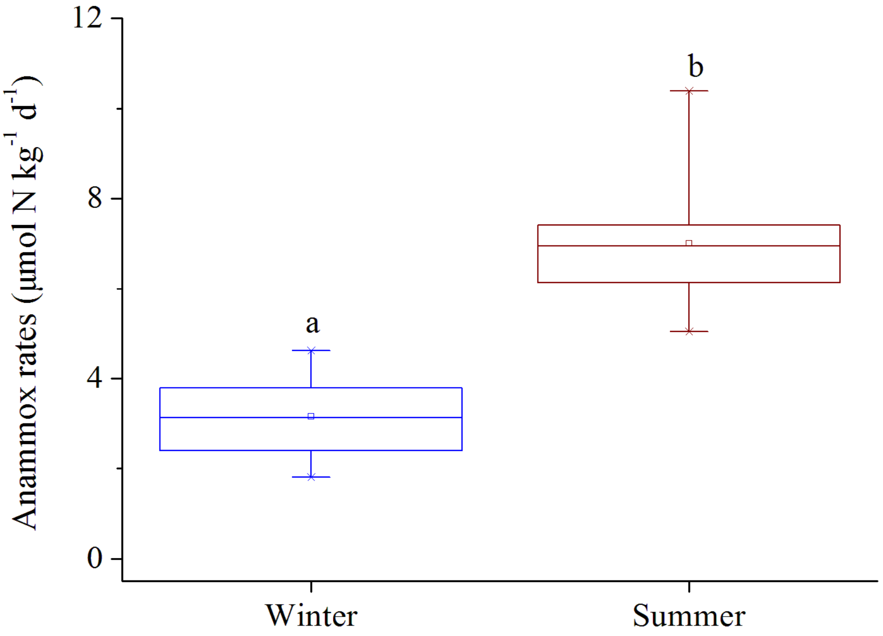

Supplementary Figure S4 Seasonal difference in anammox rates in the coastal wetlands of China. The squares represent means and solid lines represent median values. Boxes enclose the interquartile range, whiskers show the full range. The lowercase letters indicate the statistically significant difference in means of anammox rates between winter and summer seasons ($p < 0.05$).

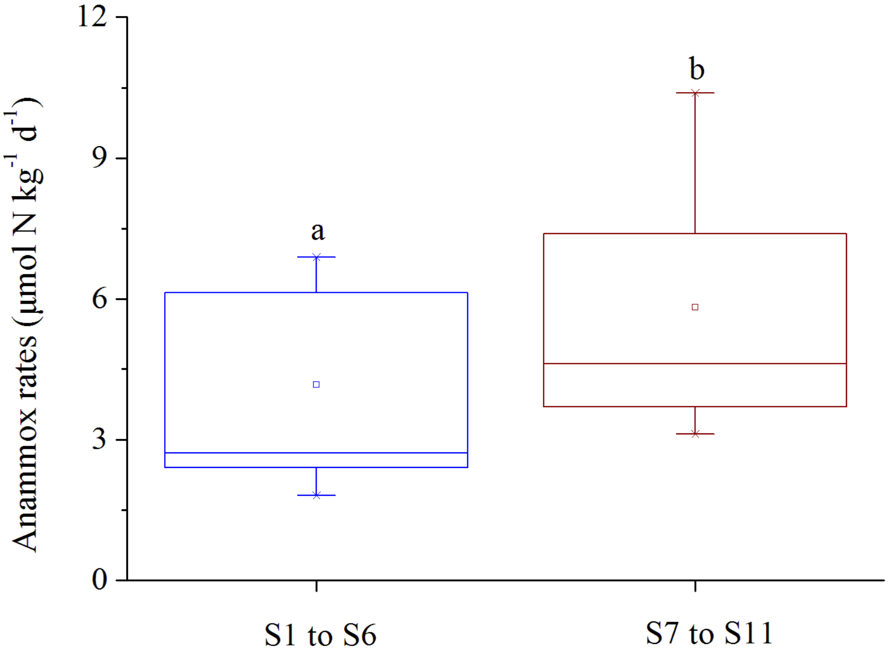

Supplementary Figure S5 Spatial difference in anammox rates in the coastal wetlands of China. The squares represent means and solid lines represent median values. Boxes enclose the interquartile range, whiskers show the full range. The lowercase letters indicate the statistically significant difference in means of anammox rates between the northern (S1 to S6) and southern (S7 to S11) sites ($p < 0.05$).

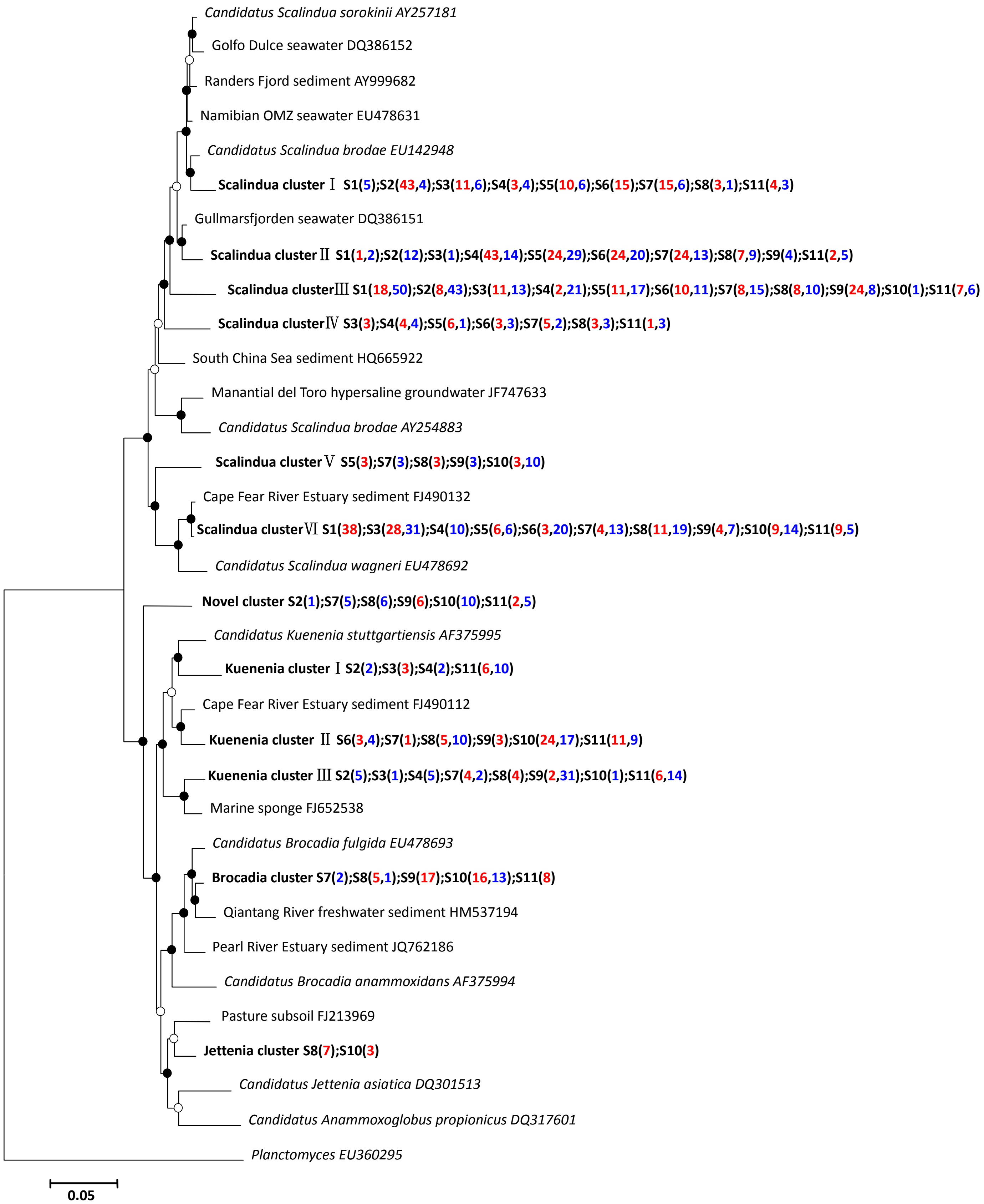

Supplementary Figure S6 Neighbour-joining phylogenetic tree showing affiliations between anammox bacteria-related 16S rRNA gene fragments in the clone libraries of samples from the study area (bold) and known anammox bacteria (italics). Clone names include the sample name and the number of times a sequence among all of the sequenced clones of summer (red) and/or winter (blue) samples. Bootstrap values (>50%) based on 1000 replications are shown with solid circle symbols, and those less than 50% are shown with open circle symbols on the corresponding nodes.

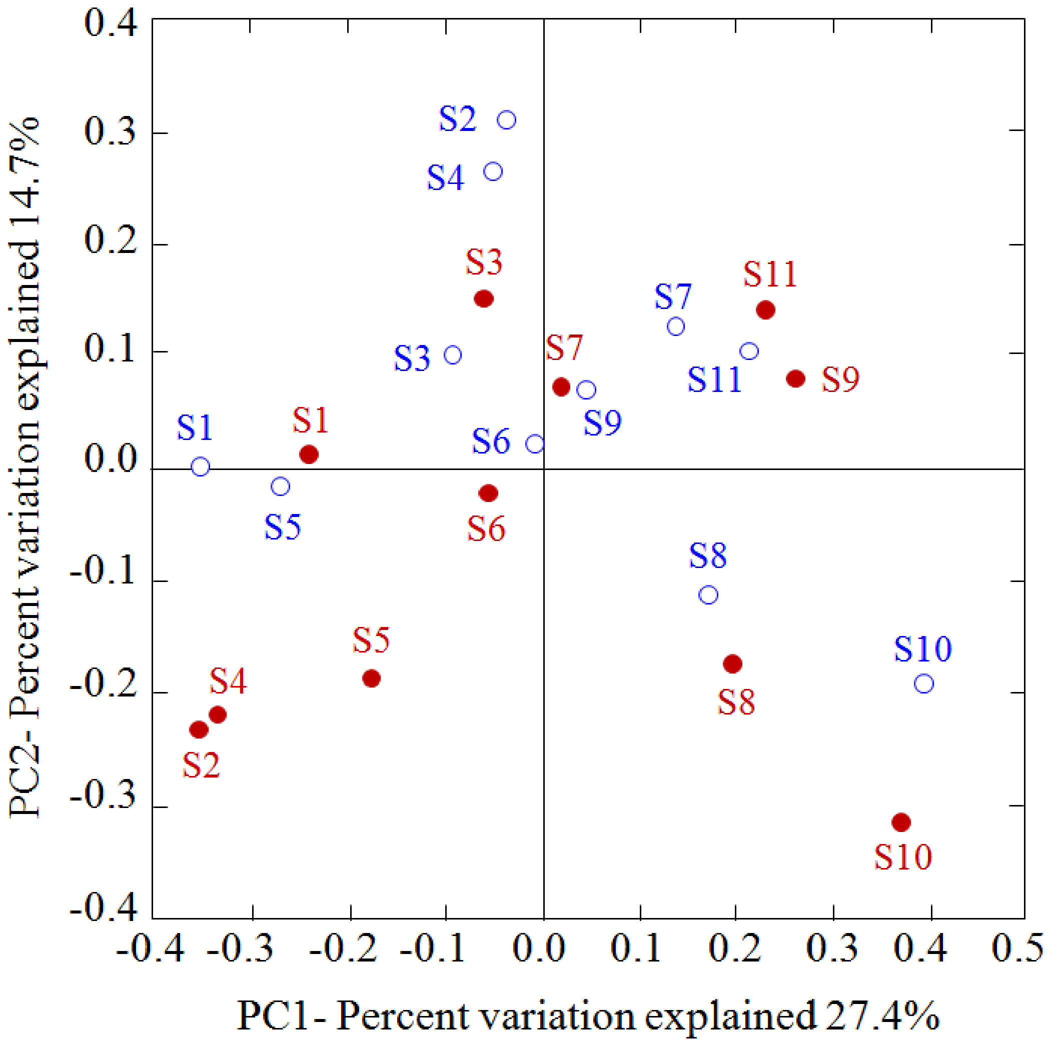

Supplementary Figure S7 The UniFrac weighted PCoA analysis of the anammox bacterial assemblages as revealed by the 16S rRNA gene sequences. Red-circle and open-circle symbols represent summer and winter samples, respectively.

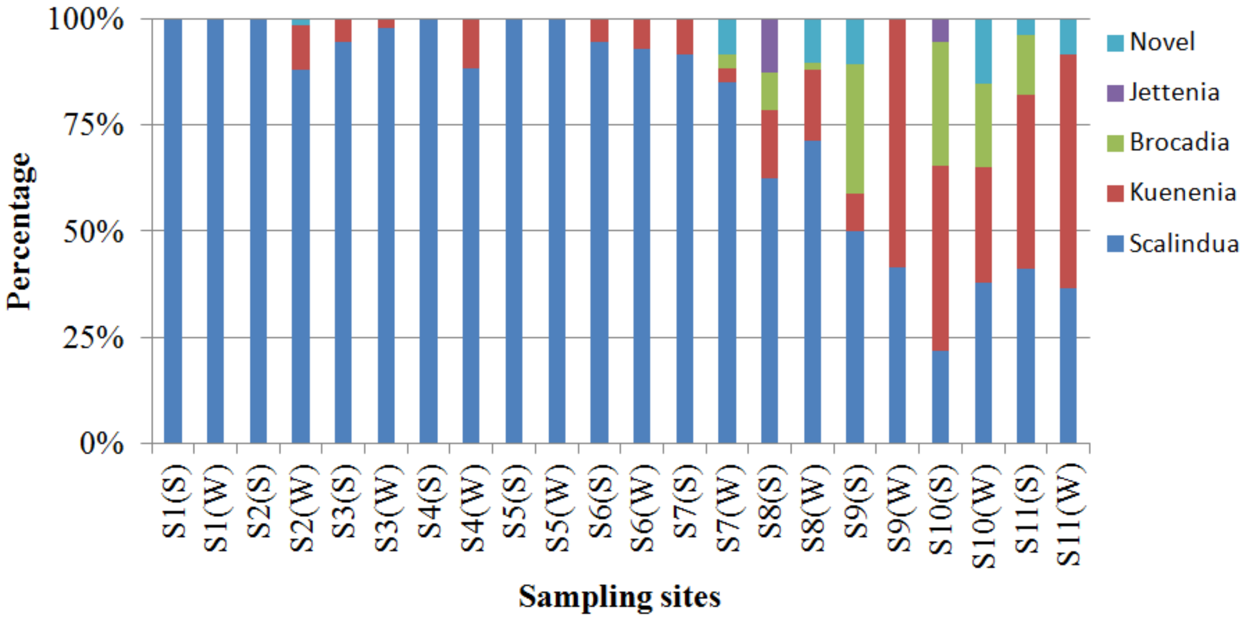

Supplementary Figure S8 The community compositions and distributions of anammox bacteria in the coastal wetland sediments of China. S and W in brackets indicate summer and winter, respectively.

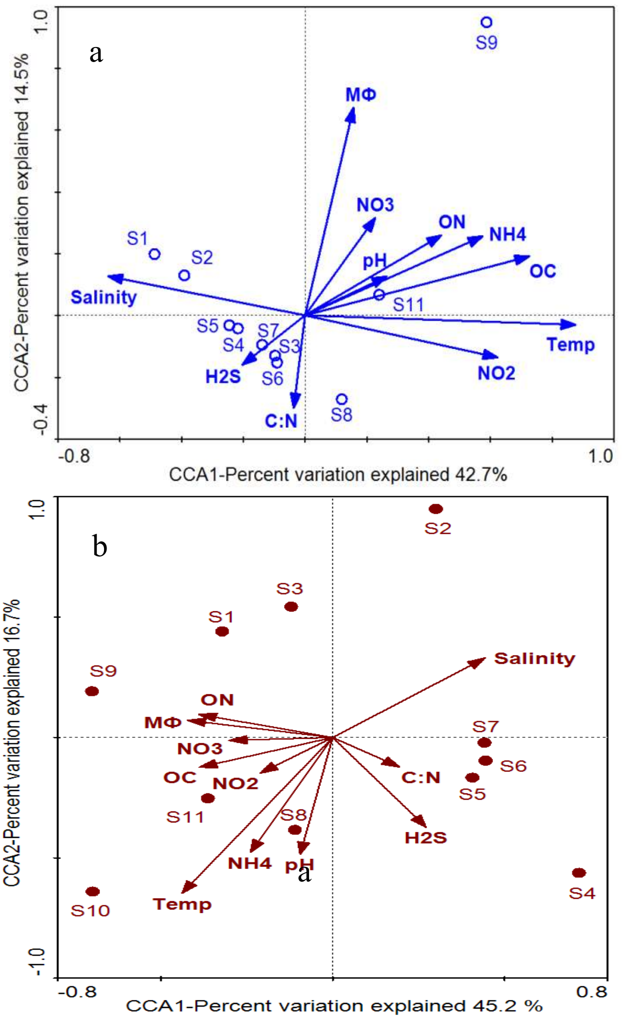

Supplementary Figure S9 CCA ordination plots for the first two principal dimensions to show the relationship between environmental factors and the anammox bacterial communities in winter (a) and summer (b). Correlations between environmental variables and CCA axes are represented by the length and angle of arrows. Temp and MΦ mean temperature and sediment mean size, respectively. This analysis shows that all the detected environmental factors, only temperature was significantly correlated to the distribution of anammox bacterial communities (p = 0.002 for winter; p = 0.038 for summer).

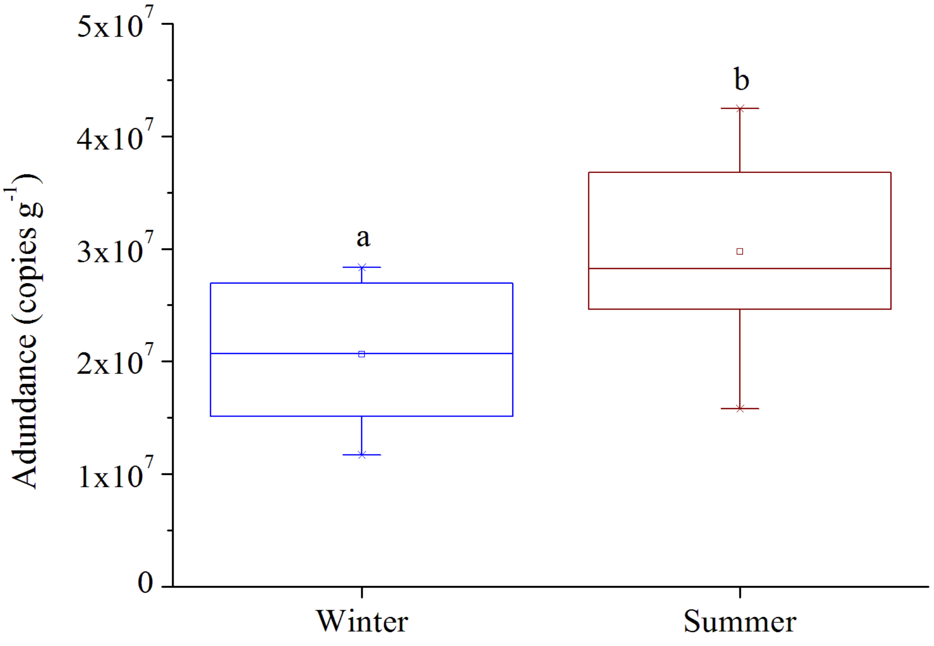

Supplementary Figure S10 Seasonal difference in anammox bacterial abundance in the coastal wetlands of China. The squares represent means and solid lines represent median values. Boxes enclose the interquartile range, whiskers show the full range. The lowercase letters indicate the statistically significant difference in means of anammox rates between winter and summer seasons ($p < 0.05$).

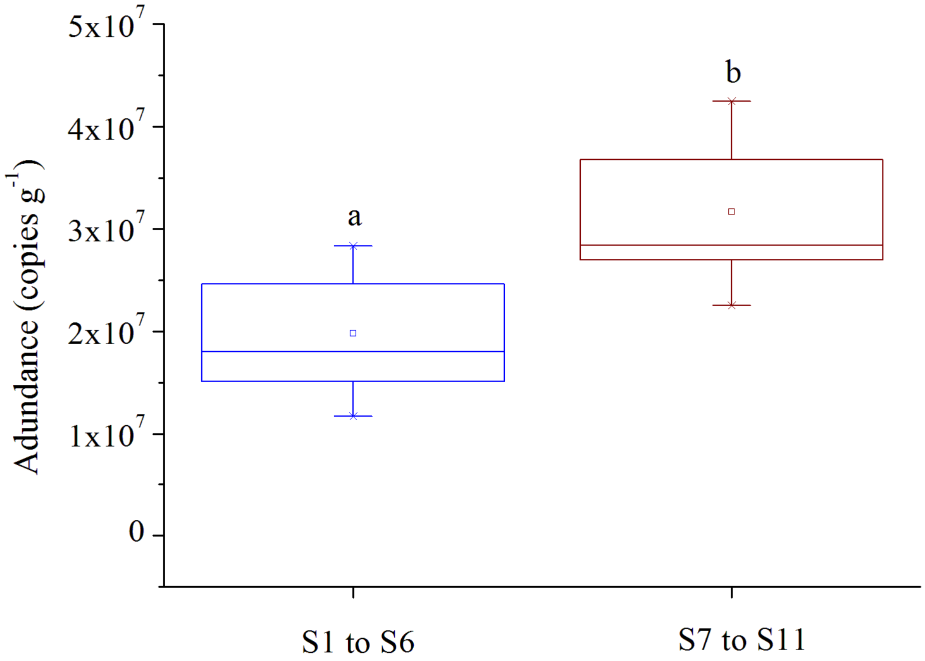

Supplementary Figure S11 Spatial difference in anammox bacterial abundance in the coastal wetlands of China. The squares represent means and solid lines represent median values. Boxes enclose the interquartile range, whiskers show the full range. The lowercase letters indicate the statistically significant difference in means of anammox rates between the northern (S1 to S6) and southern (S7 to S11) sites ($p < 0.05$).

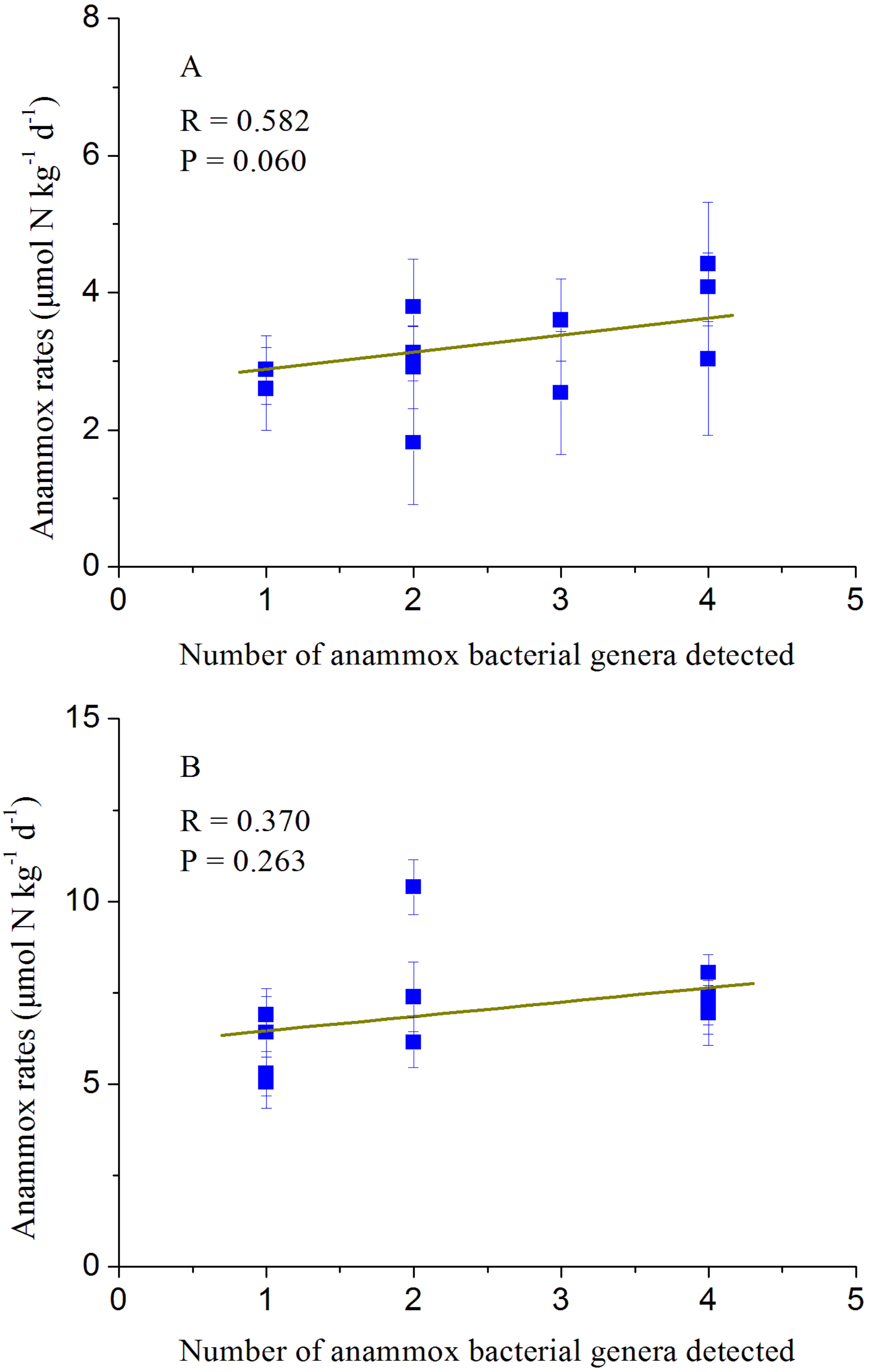

Supplementary Figure S12 Pearson's correlations of anammox rates with numbers of anammox bacterial genera detected in winter (A) and summer (B), respectively. Vertical bars indicate standard error (n = 3).

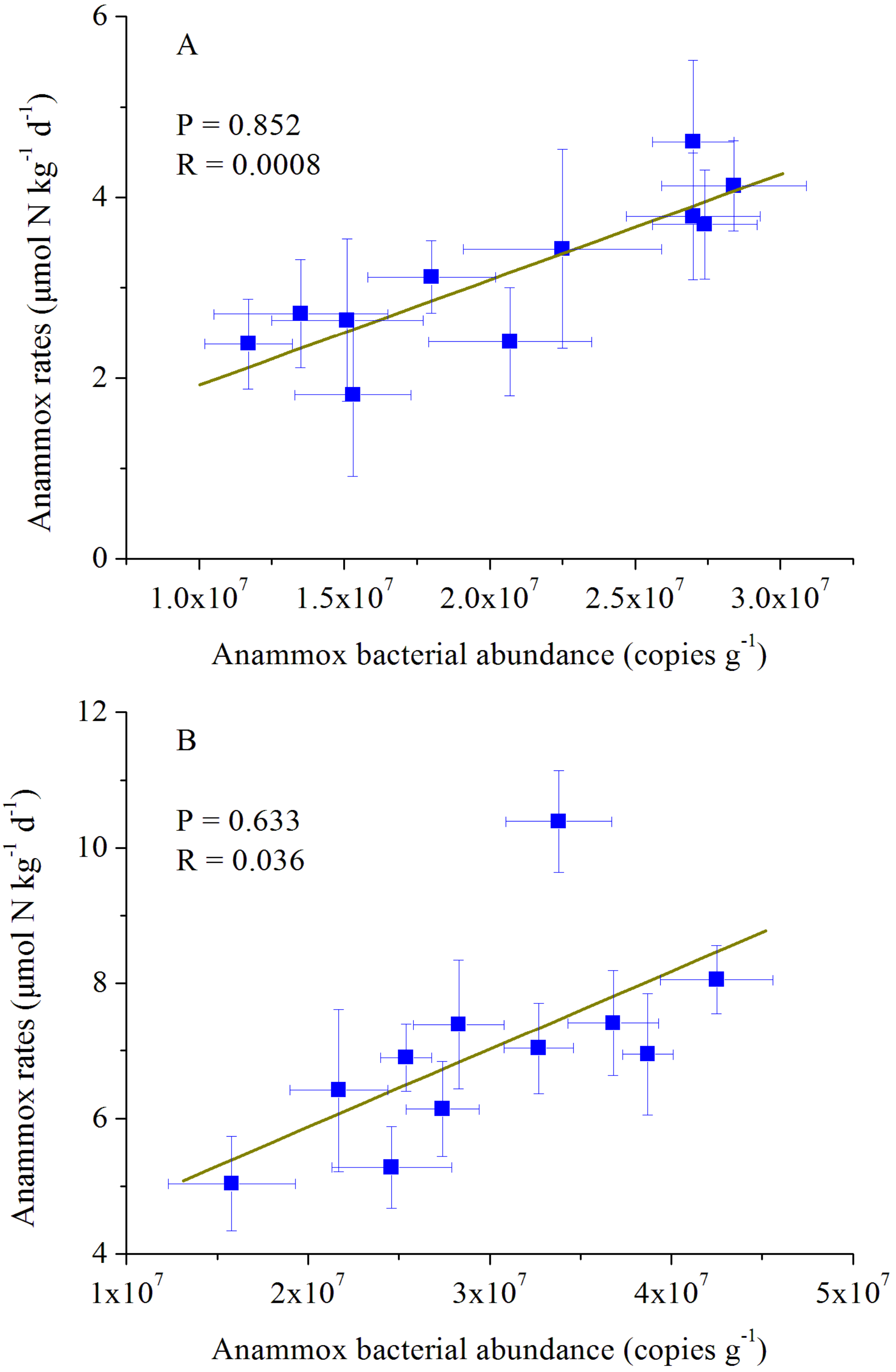

Supplementary Figure S13 Pearson's correlations of anammox rates with anammox bacterial abundance detected in winter (A) and summer (B), respectively. Vertical and horizontal bars indicate standard error (n = 3).

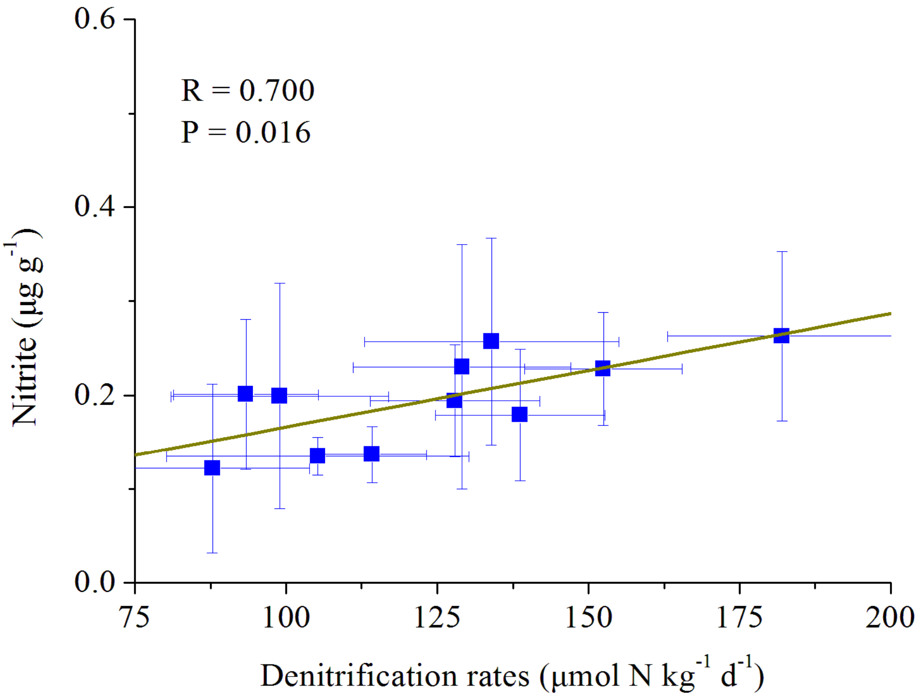

Supplementary Figure S14 Pearson's correlations of dentirification rates with sedimentary nitrite concentrations in summer sediments. Vertical and horizontal bars indicate standard error (n = 3).

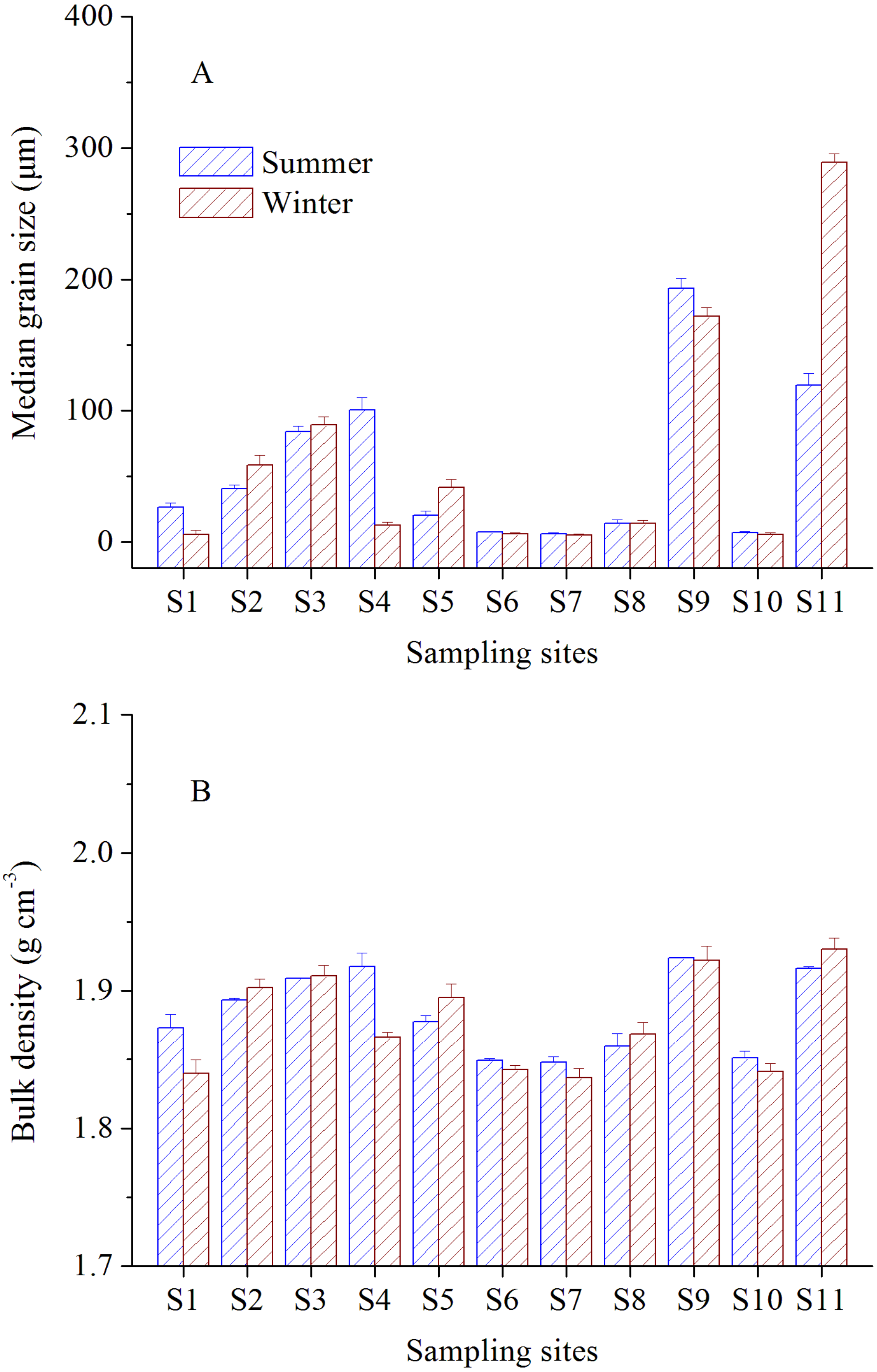

Supplementary Figure S15 Median grain size (d_{50}) of sediment (A) and bulk density (dBD) of dry sediment (B) at the study area. Dry sediment bulk density is quantified according to the relationship between d_{50} and dBD ($dBD = 2 - 0.229/d_{50}^{0.21})^3$. Average dBD values of winter and summer sediments at the study area were identical (about 1.88 g cm^{-3}). Vertical bars indicate standard error (n = 3).

Supplementary Table S1 Physio-chemical characteristics of sediments in China's coastal wetland. Data show the mean values with standard errors in parentheses (n = 3). Temp, MΦ, OC and ON represent temperature, sediment mean size, organic carbon and nitrogen, respectively.

Season	Site	Temp (°C)	Salinity (‰)	pH	MΦ (μm)	Sulfide (μmol/g)	NO_3^--N (μg/g)	NO_2^--N (μg/g)	NH_4^+-N (μg/g)	OC (%)	ON (‰)	C/N
Winter	S1	0.6(0.2)	26.4(0.3)	7.39(0.4)	56.1(5.3)	1.33(0.3)	2.07(0.3)	0.31(0.02)	5.84(1.2)	0.52(0.06)	0.36(0.05)	16.97(1.8)
	S2	1.2(0.3)	32.7(0.5)	7.61(0.5)	63.2(8.9)	17.46(1.5)	3.68(0.7)	0.29(0.08)	9.49(0.7)	0.34(0.04)	0.32(0.03)	12.91(0.7)
	S3	1.6(0.2)	28.4(0.2)	7.42(0.6)	93.9(6.2)	24.17(2.8)	2.14(0.4)	0.30(0.06)	7.83(0.8)	0.82(0.09)	0.45(0.04)	21.38(0.4)
	S4	2.1(0.3)	28.4(0.3)	7.43(0.4)	197.7(8.4)	17.22(1.7)	2.73(0.2)	0.34(0.03)	8.15(1.3)	0.45(0.13)	0.37(0.05)	14.02(2.5)
	S5	3.5(0.2)	26.4(0.4)	7.57(0.7)	37.1(4.5)	0.71(0.3)	2.41(0.3)	0.35(0.09)	5.32(0.6)	0.76(0.12)	1.06(0.14)	8.35(0.6)
	S6	5.2(0.3)	23.5(0.4)	7.13(0.5)	26.5(2.3)	0.50(0.4)	1.32(0.6)	0.46(0.12)	8.87(1.5)	0.38(0.04)	0.22(0.02)	19.82(1.3)
	S7	7.5(0.4)	26.1(0.5)	7.06(0.6)	13.3(4.3)	1.11(0.7)	1.14(0.2)	0.42(0.09)	12.29(1.3)	0.65(0.13)	0.61(0.09)	12.46(2.2)
	S8	8.2(0.3)	24.5(0.3)	7.33(0.4)	52.2(6.7)	1.87(0.5)	1.59(0.4)	0.45(0.11)	14.96(2.5)	0.56(0.03)	0.29(0.04)	22.37(1.6)
	S9	10.4(0.2)	24.4(0.4)	7.56(0.3)	240.4(7.8)	1.59(0.8)	3.55(0.3)	0.42(0.07)	14.82(1.8)	0.99(0.21)	0.89(0.11)	12.93(2.8)
	S10	11.8(0.3)	23.4(0.5)	7.66(0.5)	16.6(2.3)	7.79(1.3)	3.24(0.7)	0.45(0.06)	9.33(1.2)	0.89(0.07)	0.70(0.11)	14.99(1.3)
	S11	12.4(0.4)	25.0(0.6)	7.29(0.4)	16.6(5.7)	1.94(0.6)	1.30(0.6)	0.58(0.13)	15.53(2.7)	0.92(0.15)	1.12(0.10)	9.63(2.2)
Summer	S1	20.1(0.3)	22.5(0.4)	7.52(0.4)	8.2(2.1)	0.28(0.09)	0.96(0.3)	0.13(0.06)	12.22(1.4)	0.86(0.11)	0.77(0.17)	13.19(1.5)
	S2	20.5(0.2)	29.6(0.3)	7.47(0.6)	91.0(6.9)	1.42(0.5)	1.07(0.7)	0.20(0.04)	20.62(3.4)	0.66(0.12)	0.41(0.09)	18.89(0.9)
	S3	21.3(0.2)	30.2(0.2)	7.83(0.5)	85.6(9.2)	0.02(0.01)	1.08(0.5)	0.19(0.08)	7.11(1.7)	0.86(0.14)	0.86(0.05)	11.68(2.6)
	S4	23.2(0.3)	28.8(0.4)	7.49(0.5)	38.2(3.7)	3.92(0.4)	1.01(0.3)	0.14(0.06)	16.25(2.1)	0.78(0.06)	0.53(0.06)	17.04(1.9)
	S5	24.6(0.3)	28.1(0.3)	8.04(0.4)	42.0(8.3)	0.01(0.0)	0.94(0.4)	0.12(0.09)	10.77(1.6)	0.68(0.08)	0.92(0.11)	8.73(0.5)
	S6	26.4(0.4)	20.6(0.5)	7.91(0.3)	10.4(2.1)	0.07(0.02)	1.04(0.6)	0.19(0.05)	17.35(1.3)	0.74(0.05)	0.75(0.14)	11.43(2.1)
	S7	28.7(0.2)	24.5(0.4)	7.97(0.4)	8.1(3.4)	0.21(0.07)	1.18(0.7)	0.26(0.04)	12.71(1.8)	0.96(0.18	0.52(0.06)	21.43(2.6)
	S8	29.1(0.3)	25.1(0.5)	7.73(0.6)	58.7(7.2)	0.25(0.08)	1.13(0.3)	0.25(0.03)	39.29(2.8)	0.89(0.25)	0.42(0.09)	24.81(2.3)
	S9	30.2(0.3)	22.7(0.4)	7.51(0.6)	190.2(9.5)	0.02(0.01)	1.16(0.5)	0.17(0.06)	8.18(1.2)	0.89(0.23)	1.12(0.19)	9.32(3.2)
	S10	31.3(0.3)	22.4(0.2)	8.28(0.7)	13.7(5.3)	2.07(0.8)	1.06(0.4)	0.22(0.08)	44.58(4.7)	0.79(0.08)	0.75(0.12)	12.31(1.1)
	S11	31.5(0.5)	23.8(0.4)	7.78(0.5)	312.2(7.9)	0.37(0.12)	1.15(0.2)	0.23(0.09)	11.54(2.5)	0.85(0.12)	0.49(0.06)	20.29(2.7)

Supplementary Table S2 Pearson's correlation analyses between anammox rates and sediment characteristics (n = 11). Temp, MΦ, OC and ON represent temperature, sediment mean size, organic carbon and nitrogen, respectively; R denotes Pearson's correlation coefficients; P denotes the significant level of the correlation.

Season		Salinity (‰)	Temp (°C)	pH	MΦ (μm)	Sulfide (μmol/g)	NO_3^--N (μg/g)	NO_2^--N (μg/g)	NH_4^+-N (μg/g)	OC (%)	ON (‰)	C/N
Winter	R	-0.542	0.752**	-0.346	-0.242	-0.497	-0.351	0.667*	0.833**	0.349	0.156	0.176
	P	0.086	0.008	0.258	0.474	0.120	0.250	0.024	0.002	0.252	0.648	0.606
Summer	R	-0.458	0.607*	0.259	-0.170	-0.067	0.649*	0.692*	0.211	0.717*	-0.282	0.495
	P	0.156	0.048	0.441	0.618	0.844	0.030	0.018	0.532	0.012	0.402	0.122

Supplementary Table S3 Pearson's correlation analyses between anammox bacterial abundance and sediment characteristics (n = 11). Temp, MΦ, OC and ON represent temperature, sediment mean size, organic carbon and nitrogen, respectively; R denotes Pearson's correlation coefficients; P denotes the significant level of the correlation.

Season		Salinity (‰)	Temp (°C)	pH	MΦ (μm)	Sulfide (μmol/g)	NO_3^--N (μg/g)	NO_2^--N (μg/g)	NH_4^+-N (μg/g)	OC (%)	ON (‰)	C/N
Winter	R	-0.555	0.862**	-0.241	-0.106	-0.585	-0.260	0.759**	0.814**	0.514	0.536	-0.286
	P	0.072	0.000	0.476	0.656	0.058	0.440	0.006	0.002	0.106	0.090	0.394
Summer	R	-0.404	0.790**	0.181	0.359	-0.054	0.802**	0.856**	0.527	0.574	-0.353	0.582
	P	0.218	0.004	0.596	0.278	0.876	0.004	0.000	0.096	0.064	0.286	0.060

Supplementary Table S4 Summary of anammox bacterial biodiversity in China's coastal wetlands.

Seasons	Sites	Sequence Numbers	OTUs Numbers	Chao1 Estimate	Shannon Index	Coverage (%)
Summer	S1	57	8	8.5	1.20	94.1
	S2	67	16	16.0	2.19	100.0
	S3	52	15	16.0	2.32	93.8
	S4	60	23	23.5	2.95	98.0
	S5	59	15	15.6	2.26	96.2
	S6	58	13	13.0	2.26	100.0
	S7	61	22	23.1	2.88	95.2
	S8	59	18	20.0	2.56	90.0
	S9	53	11	11.2	1.64	98.5
	S10	66	21	21.3	2.77	98.8
	S11	60	21	23.3	2.80	90.1
Winter	S1	57	9	9.0	1.83	100.0
	S2	51	2	2.0	0.43	100.0
	S3	56	16	16.5	2.53	97.0
	S4	52	12	12.6	1.91	95.2
	S5	60	23	25.1	2.93	91.5
	S6	58	16	16.5	2.32	97.0
	S7	61	21	23.0	2.68	91.3
	S8	56	25	26.9	3.09	92.9
	S9	56	11	11.3	1.92	97.1
	S10	55	12	12.8	2.06	94.1
	S11	56	18	20.0	2.69	90.0

References in Supplementary Information

1. Cui, S.H., Shi, Y.L., Groffman, P.M., Schlesinger, W.H. & Zhu, Y.G. Centennial-scale analysis of the creation and fate of reactive nitrogen in China (1910–2010). *Proc. Natl. Acad. Sci. USA* 110, 2052-2057 (2013).
2. Zhao, D.Z., Zhao, L., Zhang, F.S. & Zhang, X.Y. Temporal occurrence and spatial distribution of red tide events in China's coastal waters. *Hum. Ecol. Risk Assess.* 10, 945–957 (2004).
3. Verstraeten, G. & Poesen, J. Variability of dry sediment bulk density between and within retention ponds and its impact on the calculation of sediment yields. *Earth Surf. Process. Landforms*, **26**, 375-394 (2001).
